# Electroporation induced changes in extracellular vesicle profile

**DOI:** 10.1080/10717544.2025.2562224

**Published:** 2025-09-22

**Authors:** M. Singh, G. Mazaheri-Tehrani, I. Martin-Fabiani, O. G. Davies

**Affiliations:** a School of Sport, Exercise and Health Sciences, Loughborough University, Loughborough, UK; b Department of Materials, Loughborough University, Loughborough, UK

**Keywords:** Extracellular vesicles, electroporation, drug delivery, loading, nanoparticle

## Abstract

Extracellular vesicles (EVs) are promising drug delivery systems (DDSs). Electroporation is widely applied in the loading of therapeutic payloads but has not been optimized for EV loading. Understanding the potential effects of electroporation on EV profile and integrity is important if they are to be applied therapeutically. In the present study, EVs were isolated and subjected to electroporation at different voltages (500–1000 mV), pulse numbers (1–3), and pulse widths (10–30 ms). Particle concentration, size distribution, polydispersity index (PDI), zeta potential (ZP), and protein concentration were analyzed and western blotting was performed to evaluate possible variations in EV surface markers. Suspension in electroporation buffer (EB) significantly reduced EV concentration, increased particle size, and reduced the ZP. Native EV profile could not be recovered following washing. Electroporation parameters (EPs) applied had variable effects on in EV profile, with reductions in surface protein concentration and a more neutral ZP observed. In conclusion, we identified that the electroporation protocol had a considerable impact on basic EV properties, which could impact their application as DDS. Further optimization of EBs and protocols is required to retain native EV profile following loading.

## 1. Introduction

Extracellular vesicles (EVs) are heterogeneous, membrane-bound nanoparticles secreted by both prokaryotic and eukaryotic cells (Kalra et al. 2016). Once thought to provide a means of removing waste, they are now well documented to function in the transfer of a variety of bioactive materials (proteins, lipids, and nucleic acids) between local and distant cells and tissues (Zeng et al. 2022). This knowledge has led to a growing interest in their application as drug delivery systems (DDSs) (Wang et al. 2022). Notably, EVs can cross biological barriers such as the blood–brain barrier and blood–lymph barrier (Elliott and He 2021). While their stability, tropism, low immunogenicity, and potential expression of anti-phagocytic ‘don’t eat me’ signals such as CD47 make them attractive natural platforms for drug delivery (Elsharkasy et al. 2020; Zeng et al. 2023). Collectively, these features offer many potential advantages over synthetic DDS, such as liposomes and lipid nanoparticles.

The application of EVs as DDS relies on the ability to efficiently and reproducibly load therapeutic molecules of choice. The loading method selected is dependent on the nature of the payload, with endogenous strategies such as genetic engineering often being applied for gene and protein loading. When dealing with small molecules and pharmaceuticals, it is common to isolate the EVs and directly apply passive or active loading strategies, depending on the molecular weight and polarity of the desired payload (Rankin-Turner et al. 2021). Several active methods function by temporarily disrupting the integrity of the lipid bilayer to facilitate transfer into the EV lumen (Rankin-Turner et al. 2021). Of these, electroporation is perhaps the most widely used. This method has been well described for cell transfection and applies a transient electrical stimulus to create temporary membranal pores through which charged hydrophilic small molecules can pass (Hood et al. 2014; Gaurav et al. 2021). Removal of the electric field leads to reformation of the pores and encapsulation of the payload (Nakamura and Funahashi 2013).

Over the past decade, electroporation has become increasingly applied in EV studies. The majority have explored nucleic acid loading (specifically, miRNA and siRNA) and small molecules such as doxorubicin (DOX) (Pomatto et al. 2019; Rankin-Turner et al. 2021). However, within these studies exists a high degree of variability in the electroporation parameters (EPs) applied (Rankin-Turner et al. 2021; Roerig and Schulz-Siegmund 2023). Furthermore, there exists some evidence to suggest the application of EPs, such as voltage, can negatively impact biophysical properties such as morphology and stability (Kibria et al. 2018; Danilushkina et al. 2023). Given the small radius of EVs, the application of a high field strength may be required to achieve sufficient membrane permeabilization, which could cause damage to the EVs and/or their cargo (Dimitrov et al. 2013; Lennaárd et al. 2022). Despite these considerations, studies documenting the effects of EPs on EV characteristics, such as morphology, stability, and function, remain limited (Kibria et al. 2018; Rankin-Turner et al. 2021). Unlike cells, EVs are non-replicative, with any damage induced by the application of an electric field having a potentially permanent effect. This could compromise their downstream therapeutic utility. For example, in lipid nanoparticles, the application of voltage can induce morphological changes, causing them to prolate or oblate (Dimova and Riske 2017). Such changes can weaken the membrane, making lipid nanoparticles more susceptible to rupture (Sadik et al. 2011). While parameters like voltage (V) and pulse duration (PD), when applied at high intensities, can generate heat from excessive energy, leading to thermal damage (Batista Napotnik et al. 2021; Kranjc Brezar et al. 2023).

Electroporation is a widely applied method used to load EVs with a variety of therapeutic payloads. Commercial transfection systems have been widely applied for this purpose (Pomatto et al. 2019; Zhang et al. 2020; Busatto et al. 2021; Pan et al. 2023). However, these systems were originally developed for the transfection of cells and are now applied for EV loading without further optimization. As such, we sought to determine the impact of a commercial electroporation buffer (EB) and loading parameters (voltage, pulse number, and pulse width) on fundamental EV properties that could impact their downstream application as a DDS.

## 2. Materials and methods

### 2.1. C2C12 cell culture and conditioned media collection

EVs used in this study were derived from C2C12 murine myoblast cells. C2C12s are a clonal cell line, offering a consistent and reproducible source of EVs. Additionally, C2C12s can be cultured in low concentrations of EV-depleted horse serum (HS) (2%), reducing the presence of residual exogenous EVs within our preparations (Lehrich et al. 2021). Passage 7 C2C12 cells (ECACC, Sigma-Aldrich, St. Louis, MO) were seeded at a density of 3.75 × 10^5^ cells/cm^2^ (Nunclon™ Delta Surface, Thermo Fisher Scientific, Oxford, UK) and cultured until confluent in growth media (GM); composed of high glucose Dulbecco’s modified Eagle’s medium (DMEM) (Sigma Life Science, Burlington, MA) supplemented with 20% fetal bovine serum (FBS) (PAN-Biotech, Minster, UK) and 1% penicillin/streptomycin (P/S) (Sigma-Aldrich, St. Louis, MO). Upon reaching confluence, GM was replaced with differentiation media (DM); composed of high glucose DMEM supplemented with 1% P/S and 2% EV-depleted HS (Sigma Life Science, Burlington, MA). EVs were depleted from HS using ultracentrifugation (UC) at 120,000 × *g* for 16 hours at 4 °C using 20 mL thick-walled polycarbonate tubes (S309156A, SLS) in the Himac CS150FNX 911097C3 Ultra Microcentrifuge with a S50A rotor (Himac, Hitachinaka, Japan). After five days in the DM, the conditioned media was collected and centrifuged at 2000 × *g* for 20 minutes to remove cells and debris before storing at −80 °C until required for EV isolation.

### 2.2. Extracellular vesicle isolation

Conditioned media was first spun at 10,000 × *g* for 30 minutes at 4 °C using an S50A rotor (S309156A, SLS) to eliminate larger particles followed by UC of the supernatant at 120,000 × *g* for 70 minutes at 4 °C. The EV pellet was resuspended in Dulbecco’s phosphate buffered saline (DPBS) (Gibco, London, UK) and stored at −80 °C.

### 2.3. Electroporation

**Figure 1.**
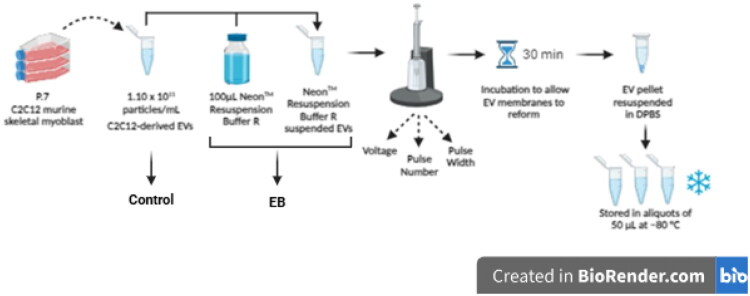
Characteristics of EB resuspended EVs compared to control EVs. (A) Particle concentration measurements determined by NTA for control and EB EVs. (B) Size distribution plot of control and EB EVs determined using NTA, with the mode size of 160.5 nm for control and 176 nm for EB. (C) *Z*-average in nm measured for control and EB using DLS. (D) Zeta potential in mV measured for control and EB using DLS. (E) Polydispersity index measured for control and EB EVs using DLS. (F) Surface protein concentration measured for control and EB EVs using BCA protein assay. Significances were calculated using unpaired *t*-test and represent as follows: *p < .05; **p < .01; ****p < .0001. *N* = 3.

**Figure 2.**
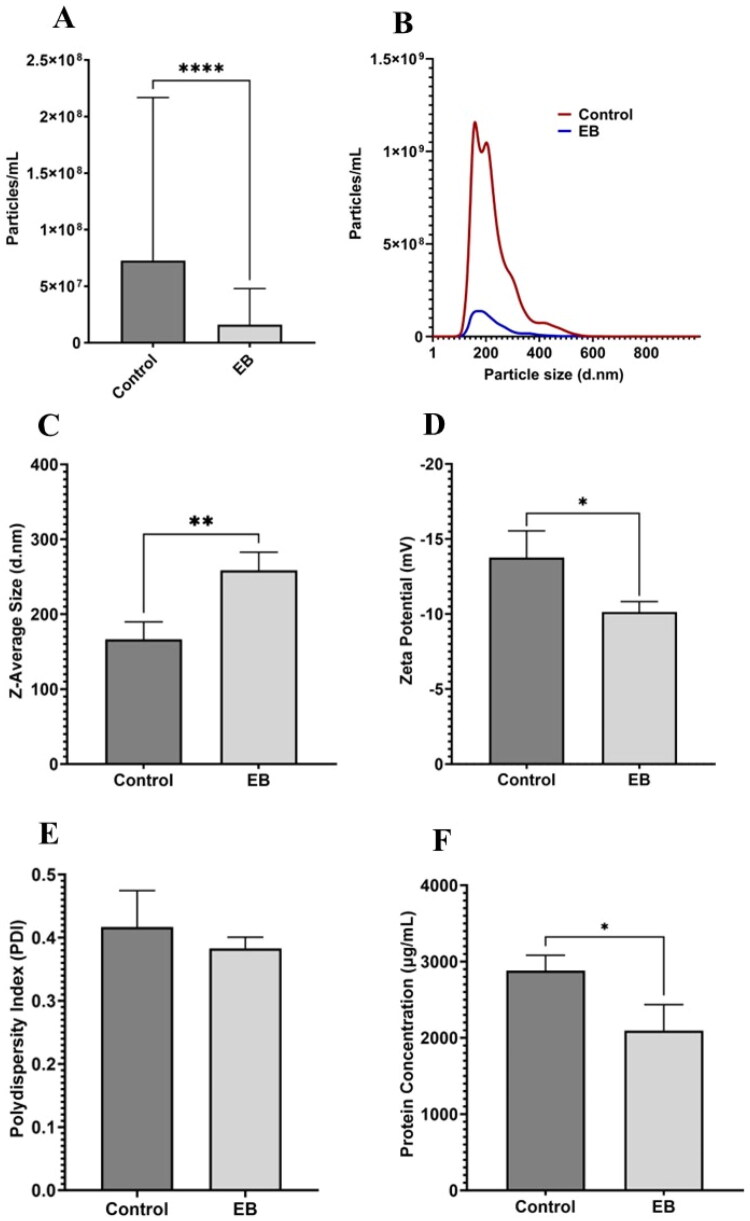
Schematic representation of the experimental procedure followed to perform electroporation on C2C12-derived EVs to assess its impact on EV characteristics. EVs electroporated at the mentioned parameters were immediately stored at –80 °C until EV characterization analysis. Illustration created on BioRender.com.

Electroporation was performed using the Neon™ Transfection System 100 µL kit (MPK10096, Invitrogen™, Thermo Fisher Scientific, Oxford, UK). EVs (3.3 × 10^11^ particles/mL) were resuspended in 100 µL of Neon™ Resuspension Buffer R (1:3) (MPK10096R, Thermo Fisher Scientific, Oxford, UK) containing 3 mL Neon™ Electrolytic Buffer E2 (MPK10096E, Thermo Fisher Scientific, Oxford, UK) (Table 1). EVs in suspension were electroporated at voltages of 500 mV, 750 mV, and 1000 mV. The process was repeated for pulse number (1, 2, and 3 pulses) and pulse width (10, 20, and 30 ms). EVs in DPBS not subjected to electroporation served as a control. EVs resuspended in Neon™ Resuspension Buffer R using a 1:3 dilution, but not subject to electroporation were used as a negative control and referred to as EB throughout (Figure 1). All samples were transferred into 100 µL of fresh DPBS and incubated for 30 minutes at RT to allow for the membrane reclosure before storing at −80 °C (Figure 2).

**Table 1.** Information on electroporation parameters applied to C2C12-derived EVs.

Electroporation condition	Neon™ resuspension buffer R	Applied parameters			
Voltage	100 µL	Voltage (mV)	500	750	1000
		Pulse number	2 pulses	2 pulses	2 pulses
		Pulse width (ms)	30	30	30
Pulse number	100 µL	Voltage (mV)	750	750	750
		Pulse number	1	2	3
		Pulse width (ms)	30	30	30
Pulse width	100 µL	Voltage (mV)	750	750	750
		Pulse number	2 pulses	2 pulses	2 pulses
		Pulse width (ms)	10	20	30
Electroporation buffer (EB)	100 µL	Voltage (mV)	–	–	–
		Pulse number	–	–	–
		Pulse width (ms)	–	–	–

### 2.4. Quantification of extracellular vesicles

Particle size distribution and concentration were analyzed using the Nanosight LM-10 (Malvern Instruments Ltd., Malvern, UK) equipped with a 405 nm laser. Data was analyzed using NTA 3.2 Dev Build 3.2.16 software. EV samples were diluted in DPBS (1:100) before loading into the sample chamber using an automated syringe pump set up to capture 6× measurements, at 30-s each run (*n* = 3 technical replicates). To achieve a strong signal and minimize background noise, the camera level was set at 12. Analysis was performed with a screen gain set at 10 and a threshold at 7. The temperature was kept at a constant of 22 °C.

### 2.5. Size distribution and zeta potential analysis

Particle size distribution and zeta potential (ZP) measurements were analyzed by dynamic and electrophoretic light scattering (DLS-ELS, Zetasizer Ultra, Malvern Panalytical, Malvern, UK). DTS1070 disposable folded capillary cells were washed with isopropanol and deionized water and dried with N_2_. EV samples (2.3 × 10^11^ particles/mL) were diluted in DPBS (1:21) to load 1.1 × 10^11^ particles/mL into the capillary cell. Size distribution was measured with an equilibrium time set at 30 s and a pause of 50 s. Three repeats were carried out for each sample. Polydispersity index (PDI) was calculated via cumulant analysis of the average correlation function. ZP measurements were carried out in monomodal mode at a voltage of 50 mV using the Smoluchowski approximation. ZS Xplorer 1.0.0.436 software was used for data capture and analysis. Temperature was kept at a constant of 22 °C.

### 2.6. Protein quantification

EV surface protein was quantified using the Pierce™ BCA protein assay kit (Thermo Fisher Scientific, Oxford, UK) according to the manufacturer’s instructions (Pierce, Rockford, IL). Briefly, 50 μL of each sample was diluted (1:1) using DPBS and loaded into a 96-well plate (Corning Costar, Ewloe, UK) in triplicates. This was followed by addition of 200 μL of the BCA copper complex solution. The plate was shaken for 30 seconds and incubated at 37 °C for 30 minutes (*n* = 3 technical replicates). Absorbance was measured at 562 nm using the Varioskan Flash 4.00.53 microplate reader (Thermo Fisher Scientific, Oxford, UK). The software used for data analysis was SkanIt Software 2.4.5 RE.

### 2.7. Western blotting for EV-associated markers

The presence of EV-associated proteins CD9 and Annexin A2 were assessed using western blot. EV samples were lysed using 4X Laemmli sample buffer (SB4X) (Bio-Rad, Kidlington, UK) containing β-mercaptoethanol, distilled water, and lysis buffer. Samples were boiled for five minutes at 98 °C to ensure lysis of protein bonds. EV samples and cell lysate were loaded at a concentration of 8 µg and electrophoresis performed on a 4–15% Mini-PROTEAN pre-cast gel (Bio-Rad, Kidlington, UK). Proteins were transferred onto polyvinylidene fluoride (PVDF) membranes (Fisher Scientific, Loughborough, UK), washed with 1× tris-buffered saline, 0.1% Tween^®^ 20 (Millipore, Feltham, UK) (TBST) before blocking in EveryBlot blocking buffer (Bio-Rad, Kidlington, UK) for five minutes at room temperature. Membranes were washed in TBST before incubating in EveryBlot blocking buffer containing primary antibodies overnight at 4 °C (see Table 2 for antibody conditions). Following overnight incubation, the membranes were washed in TBST before incubating with horseradish peroxidase (HRP)-conjugated secondary antibodies suspended in EveryBlot blocking buffer for 60 minutes. Membranes were incubated with Clarity™ Western ECL substrate (Bio-Rad, Kidlington, UK) in the dark for five minutes before their detection using the ChemiDoc XRS+ system (Bio-Rad, Kidlington, UK) and Image Lab 3.0.1 software (Life Science Research, Bio-Rad, Kidlington, UK) for analysis (*n* = 3 biological replicates).

**Table 2.** Information on primary and secondary antibodies used for western blot analysis.

Primary antibody	Product code and supplier	Source	Dilution	Secondary antibody	Product code and supplier	Dilution
Anti-Annexin A2	Abcam (ab41803)	Rabbit	1:2000	Anti-Rabbit IgG	Cell Signaling Technology (Danvers, MA) (7074)	1:3000
Anti-Calnexin	Abcam (ab22595)	Rabbit	1:1000	Anti-Rabbit IgG	Cell Signaling Technology (Danvers, MA) (7074)	1:3000
Anti-CD9	Abcam (ab92726)	Rabbit	1:1000	Anti-Rabbit IgG	Cell Signaling Technology (Danvers, MA) (7074)	1:2000

### 2.8. Data analysis

Statistical analysis was performed using an unpaired *t*-test and one-way ANOVA followed by Tukey’s post hoc test with GraphPad Prism 9.5.1 (San Diego, CA). Statistical significance is illustrated as follows: *p < .05; **p < .01; ***p < .001; ****p < .0001. Graphs were composed using GraphPad Prism 9.5.1 (San Diego, CA).

## 3. Results

### 3.1. Effect of electroporation buffer on extracellular vesicle characteristics

Initial experiments analyzed the effects of EB on the mean particle concentration hydrodynamic size of EVs and compared this with the control using NTA and DLS. Suspending EVs in Neon Transfection Buffer had a significant effect on all measurable characteristics assessed (i.e. particle concentration, size distribution, PDI, ZP, and protein concentration). A noticeable decrease was observed in mean particle concentration for EB EVs (1.59 × 10^7^ particles/mL) compared to control (7.27 × 10^7^ particles/mL) (*p* < .0001) along with a significant increase in the hydrodynamic size for EB (NTA: 258.70 nm, DLS: 265.22 nm) relative to control (NTA: 166.30 nm, DLS: 173.30 nm) (*p* = .0090) (Figure 1(A–C)). Suspension in EB resulted in a significantly less negative ZP (−10.14 mV vs. −14.10 mV, *p* = .032) (Figure 1(D)). No significant difference was observed in sample PDI (Figure 1(E)). A significant decrease in surface protein concentration was observed for EB EVs relative to control (*p* = .0266) with protein concentrations reducing from 2879 µg/mL for control, to 2095 µg/mL for EB (Figure 1(F)).

### 3.2. Can EV profile be recovered after washing?

**Figure 3.**
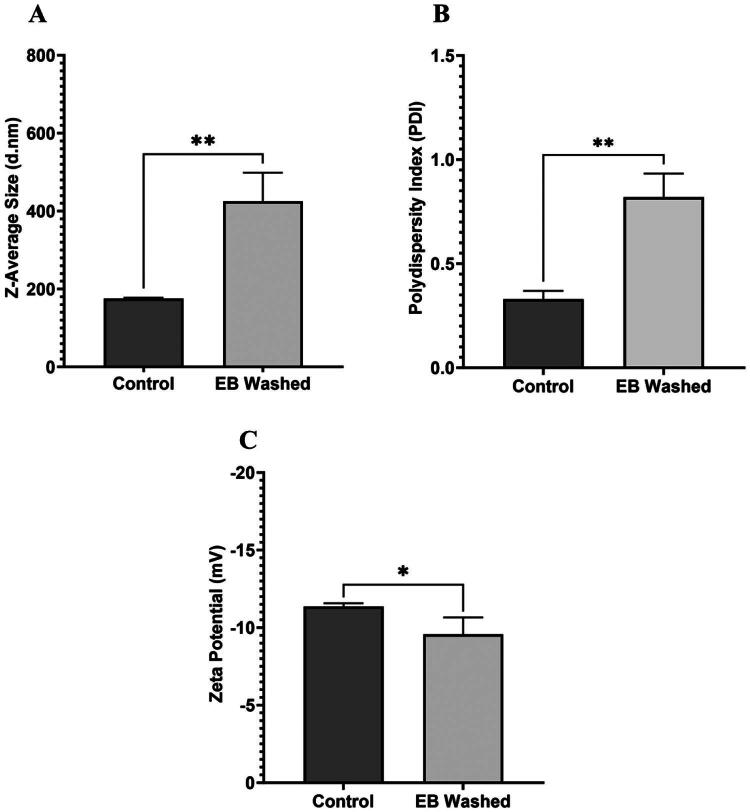
Particle size, polydispersity index, and zeta potential of EB and control EVs using DLS post washing in DPBS. (A) *Z*-average of control and EB EVs post washing in DPBS determined using DLS. (B) Polydispersity index of control and EB EVs post washing in DPBS determined using DLS. (C) Particle zeta potential of control and EB EVs post washing in DPBS determined using DLS. Significances were calculated using unpaired *t*-test and are represented as follows: *p < .05; **p < .01. *N* = 3.

Given that the suspension of EVs in EB significantly altered EV profile, we applied a DPBS washing step to determine if the native profile could be recovered. Native EV size and concentration could not be recovered after washing, with a notable increase in size from 176 nm for control EVs to 425.70 nm for EB washed EVs (*p* = .0041) (Figure 3(A)). This size increase was reflected in the PDI measurement for EB, which was also increased from 0.32 to 0.82 (*p* = .0021) (Figure 3(B)). Similarly, ZP measurements showed an increase for EB, relative to control (i.e. −11.37 mV to −9.56 mV) (*p* = .0492) (Figure 3(C)).

Given that native EV profile could not be recovered, we chose to compare the effect of EP (voltage, pulse number, and pulse width) against EVs that had been exposed to EB and washed in DPBS.

**Figure 4.**
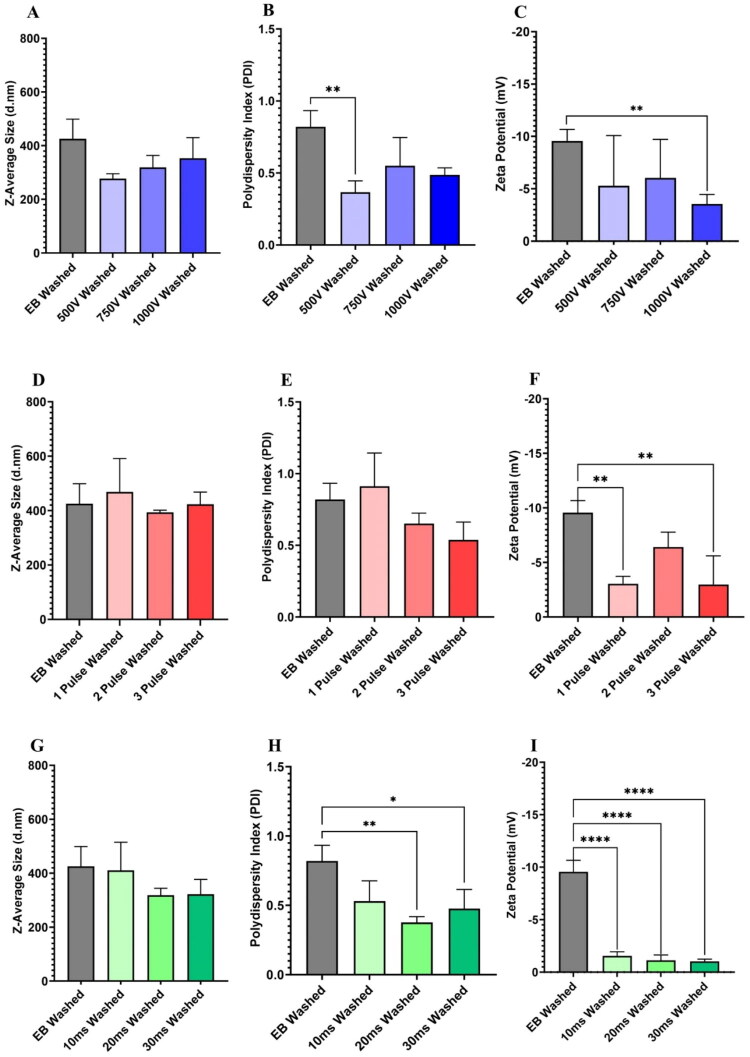
Particle size, polydispersity index, and zeta potential of EB and electroporated EVs using DLS post washing in DPBS. (A) *Z*-average of EB EVs and EVs electroporated at varying voltages post washing in DPBS determined using DLS. (B) Polydispersity index of EB EVs and EVs electroporated at varying voltages post washing in DPBS determined using DLS. (C) Zeta potential of EB EVs and EVs electroporated at varying voltages post washing in DPBS determined using DLS. (D) *Z*-average of EB EVs and EVs electroporated at varying pulse numbers post washing in DPBS determined using DLS. (E) Polydispersity index of EB EVs and EVs electroporated at varying pulse numbers post washing in DPBS determined using DLS. (F) Zeta potential of EB EVs and EVs electroporated at varying pulse numbers post washing in DPBS determined using DLS. (G) *Z*-average of EB EVs and EVs electroporated at varying pulse widths post washing in DPBS determined using DLS. (H) Polydispersity index of EB EVs and EVs electroporated at varying pulse widths post washing in DPBS determined using DLS. (I) Zeta potential of EB EVs and EVs electroporated at varying pulse widths post washing in DPBS determined using DLS. Significances were calculated using ANOVA and are represented as follows: *p < .05; **p < .01; ****p < .0001. *N* = 3.

While a decrease was observed in particle size following exposure to 500, 750, and 100 V, this decrease was not significant (Figure 4(A)). Five hundred volts produced a significant decrease in particle PDI (*p* = .0095). A significant decrease in ZP was observed following the application of 1000 V, with a 68.79% reduction observed (*p* = .0091) (Figure 4(B,C)). Pulse number did not result in changes to particle size. The application of one pulse (*p* = .0049), and three pulses (*p* = .0046) caused a significant change in ZP relative to EB, with particles becoming more neutral (Figure 4(F)). A similar effect on particle size was observed for pulse width (Figure 4(G)). The application of 20 ms (*p* = .0085) and 30 ms (*p* = .0350) produced significant decreases in PDI (Figure 4(H)). While the application of all pulse widths led to a reduction in ZP with particles again becoming more neutral (*p* < .0001) (Figure 4(I)).

**Figure 5.**
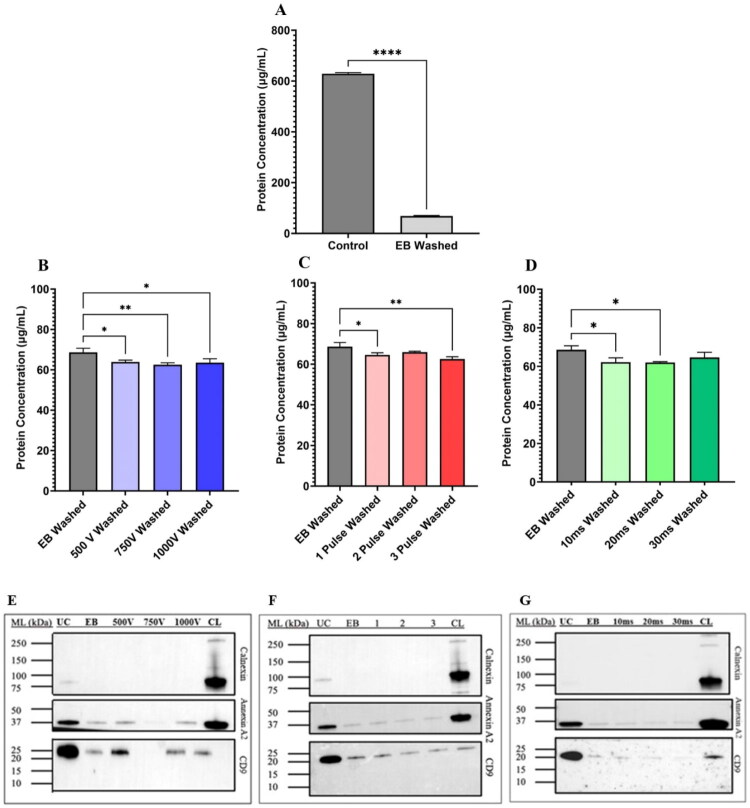
Surface protein concentration of control and EB EVs using Pierce™ BCA protein assay post washing in DPBS and the effect of applying electroporation parameters on the presence of EV-associated protein markers using western blot. (A) Surface protein concentration of EB and electroporated EVs using the Pierce™ BCA protein assay post washing in DPBS. (B) Surface protein concentration of EB EVs and EVs electroporated at varying voltages post washing in DPBS. (C) Surface protein concentration of EB EVs and EVs electroporated at varying pulse numbers post washing in DPBS. (D) Surface protein concentration of EB EVs and EVs electroporated at varying pulse widths post washing in DPBS. (E) Western blot indicating presence of EV markers Annexin A2, CD9, and negative marker calnexin in control, EB, and EVs electroporated at varying voltages. (F) Western blot indicating presence of EV markers Annexin A2, CD9, and negative marker calnexin in control, EB, and EVs electroporated at varying pulse numbers. (G) Western blot indicating presence of EV markers Annexin A2, CD9, and negative marker calnexin in control, EB, and EVs electroporated at varying pulse widths. Significances were calculated using unpaired *t*-test and one-way ANOVA and are represented as follows: *p < .05; **p < .01; ****p < .0001. *N* = 3.

Similar to hydrodynamic size, PDI, and ZP, suspension of EVs in EB significantly altered EV surface protein concentration. We recorded a 89.02% decrease in protein concentration for EB washed EVs (68.67 µg/mL) when compared with the control (628.8 µg/mL) (*p* < .0001) (Figure 5(A)). Given that native EV profile could not be recovered, we chose once again to compare the effect of EP (voltage, pulse number, and pulse width) against EVs that had been exposed to EB and washed in DPBS.

Application of 500 V (*p* = .0321), 750 V (*p* = .0081), and 1000 V (*p* = .0227) caused decreases in surface protein concentration relative to EB washed (Figure 5(B)). Pulse number caused decreases in surface protein concentration on application of one pulse (*p* = .0229) and three pulses (*p* = .0020) (Figure 5(C)). Similarly, application of 10 ms (*p* = .0220) and 20 ms (*p* = .0197) produced significant further decreases in protein concentration relative to EB washed (Figure 5(D)). A clear decrease was observed for Annexin A2 and CD9 in all samples suspended in EB relative to control. It was not possible to determine whether the application of EP had any additional impact by western blot (Figure 5(E–G)).

## 4. Discussion

EVs are an emerging DDS that offer many advantages over synthetic approaches (e.g. liposomes). Over the past decade, electroporation has become increasingly applied as a method to load EVs with therapeutic payloads including siRNAs and small molecule drugs (Kibria et al. 2018; Rankin-Turner et al. 2021). However, studies documenting the effects of EPs on EV profile remain limited and loading parameters often inconsistent (Kibria et al. 2018; Rankin-Turner et al. 2021). The present study sought to evaluate the impact of a widely used commercial EB and process variables (buffer, voltage, pulse number, and pulse width) on EV profile to define changes that could potentially impact their downstream application as a DDS.

We identified a considerable reduction in EV concentration upon suspension in EB, accompanied by an increase in EV size (2.42-fold increase). Our findings also showed a significant decrease in protein concentration, along with an increase (less negative) in ZP upon suspension in EB. This trend remained consistent when washing the EVs to remove EB (Hyder et al. 2020). These findings suggest that a commercial EB, commonly applied for cell transfection, may be suboptimal for EV loading and could negatively impact downstream applications as DDS (Pomatto et al. 2019; Zhang et al. 2020; Busatto et al. 2021; Pan et al. 2023). Increases in particle size will impact biodistribution and activate immune evasion (Liu et al. 2022; Priedols et al. 2023). An increased size may also affect their ability to cross biological barriers, which is crucial for effective drug delivery (Murphy et al. 2019; Liu et al. 2022). From the limited studies exploring the effect of electroporation on EVs, a common observation reported is aggregation (Lamichhane et al. 2015; Johnsen et al. 2016). Hence, the trends observed in our study may also be indicative of electroporation-induced EV aggregation and/or fusion (Lamichhane et al. 2015). While findings from our study identify the EB as the primary cause, it is difficult to comment precisely on how buffer composition is driving these observations, due to the proprietary nature of the buffer used (Neon™ transfection buffer). Generally, EB formulations typically comprise of a mixture of ion, salts and sugars (Sherba et al. 2020). Salts in EB can influence the conductivity of the solution, which impacts the distribution of the electric field across the membrane (Sherba et al. 2020). Changes in salt concentration have previously been shown to alter the ZP of charged nanoparticles, which could lead to aggregation and the resulting masking of surface proteins observed in the present study (Bohinc et al. 2023). Sugar constituents, such as d-glucosamine, sucrose, glucose, trehalose, and dextrose, are often reported in buffers and function in maintaining the stability of cell membranes upon the application of electric stress (High Efficiency Electroporation Buffer 2007; Igawa et al. 2014; Konov et al. 2014; Vitkova et al. 2021). While the inclusion of sugars such as trehalose (25 mM) in PBS based EV storage buffers has been shown to reduce EV aggregation and preserve integrity (Bosch et al. 2016). The concentration of sugars applied could directly impact lipid membranes, influencing nanoparticle interactions (Hasan et al. 2022). As such, commercial buffers such as the Neon™ transfection buffer may be suboptimal for the loading of EVs. However, given that the present study applied C2C12 murine myoblast EVs as a high-throughput model system, further investigations are required to determine if these outcomes are applicable to EVs from other cell sources, particularly other vesicle sources those most prevalently applied in drug delivery studies such as mesenchymal stem cell (Rehman et al. 2022; Reddy et al. 2023), HEK293T (Kim et al. 2022; Zhu et al. 2022; Nguyen Cao et al. 2023), RAW264.7 (Tang et al. 2023; Zheng et al. 2023), and milk EVs (Gong et al. 2023; Guo et al. 2023). Given the limitations of working with a proprietary – albeit widely used – EB, we also identify a need for future studies to determine the impact of EB constituents on EV profile. Notably, in addition to the effects of EB, aggregation may be further induced through the application of EV isolation methods such as UC, which is commonly used and was applied in the present study (Ahmadian et al. 2024). As such, a move toward methods that do not apply an additional external force could potentially mitigate some of the aggregation observed.

We next sought to determine the impact of active EPs (voltage, pulse number, and pulse width) on EV profile. Previous studies have demonstrated that the formation of membrane pores by electroporation can result in lipid peroxidation, damage to membrane-embedded proteins and apoptosis (Batista Napotnik et al. 2021; Peng et al. 2024). Unlike cells, EVs are static units that do not have the capacity to replace components damaged during the electroporation process. Consequently, changes caused during electroporation could have irreversible effects that potentially compromise their therapeutic utility. To date, minimal research has been published detailing the effects of EP on basic EV properties. The application of lower pulse numbers has been recommended in some EV studies. However, this is often coupled with a less efficient loading efficiency (LE) (Pomatto et al. 2019). Previous studies have observed the application of increasing pulse widths can in induce rupture, fusion, or fragmentation in lipid nanoparticles (Perrier et al. 2017; Maoyafikuddin and Thaokar 2023). Notably, the present study did not observe any significant changes in vesicle size. Rather, most changes were related to a reduction in surface protein concentration and more neutral ZP following the application of EP. Since the observed reduction in surface protein concentration was not accompanied by an increase in particle size, it is unlikely this results from the masking of proteins caused by EV aggregation and further studies are required to determine whether the changes observed negatively impact EV biodistribution and uptake (Liu and Wang 2023). Similarly, a more neutral surface charge may also impact many properties related to a vesicle’s application as a DDS. One of the most fundamental changes relates to a vesicles ability to adsorb proteins in the formation of a corona, which is a major parameter governing half-life, biodistribution, and clearance (Mitchell et al. 2021). Surface charge is also a primary factor governing cellular uptake, with more highly charged particles being more efficiently internalized (Zhang et al. 2018).

## 5. Conclusions

Electroporation is commonly applied in the active loading of EVs with therapeutic payloads. However, protocols applied have been adapted from cell transfection studies with minimal consideration of their effects on basic EV properties. The present study documented the impact of a widely utilized commercial EB on vesicle profile and described the effects of EPs (voltage, PN, and PW) on vesicle surface protein concentration and ZP. Such changes have the potential to negatively impact any downstream application as a DDS. Findings from the present study outline a need to conduct further investigators to determine the impact of translational impact of these observations in biological systems and to optimize electroporation protocols for EV studies.

## Supplementary Material

Supplementary Information.docx

## Data Availability

The datasets generated during and/or analyzed during the current study are available from the corresponding author on reasonable request.
