# Supplementary material for: Electroporation induced changes in extracellular vesicle profile
Source: Drug Deliv. 2025 Sep 22;32(1):2562224. doi: 10.1080/10717544.2025.2562224 (PMC12456048; doi:10.1080/10717544.2025.2562224)
Supplement: Supplementary Information.docx [file IDRD_A_2562224_SM0667.docx]

**Supplementary Information**


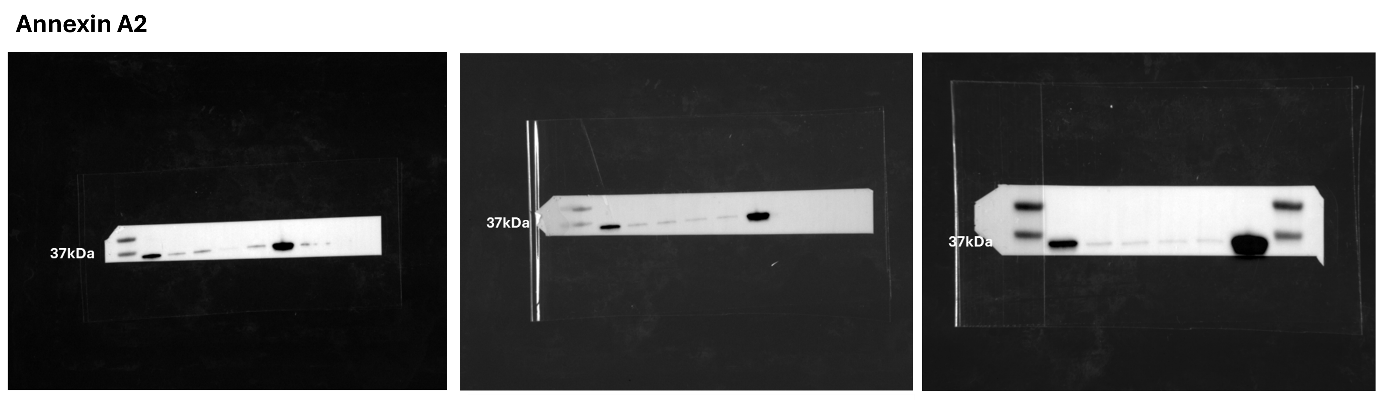


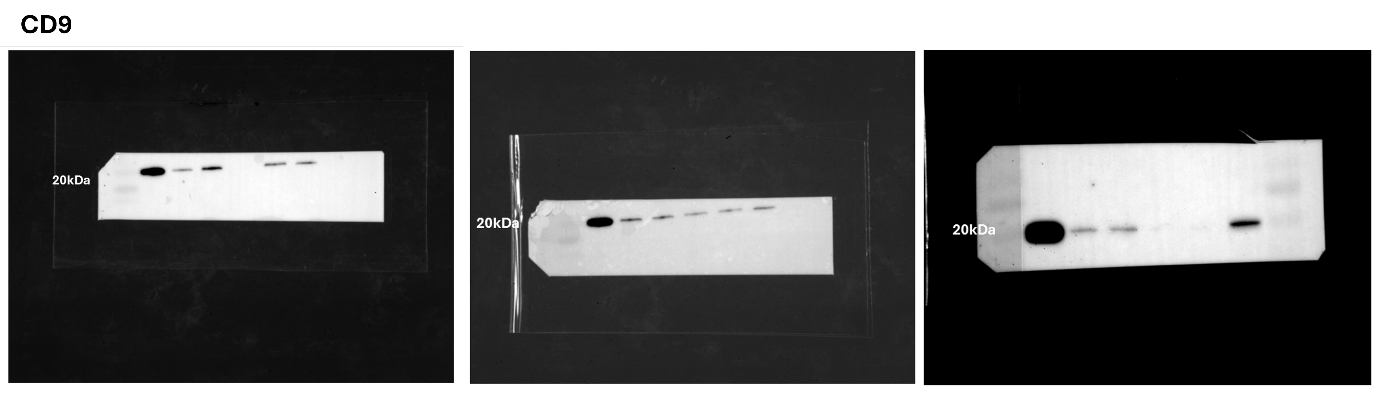


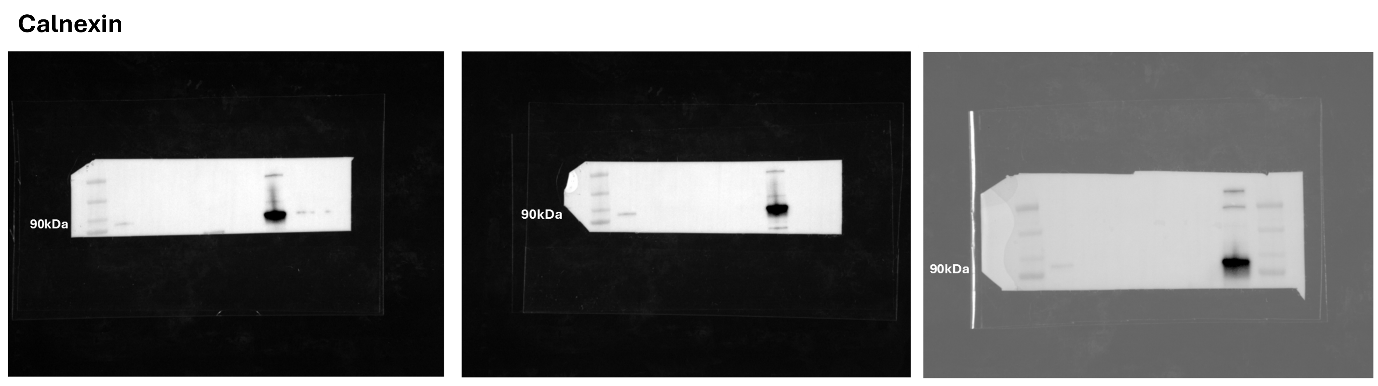


**Figure S1.** Unprocessed Western blot images for Annexin A2, CD9 and Calnexin

**A**


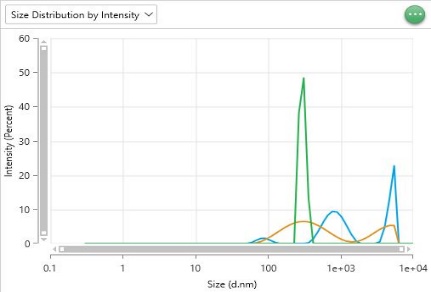

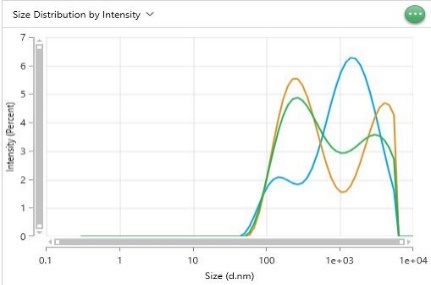

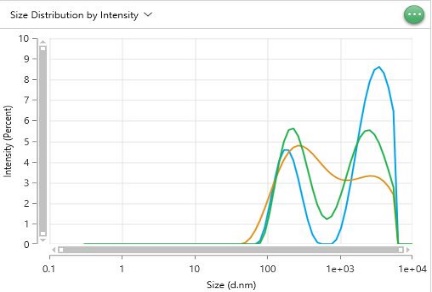

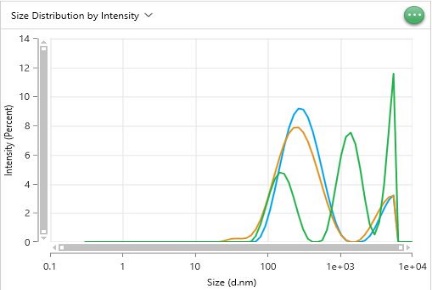

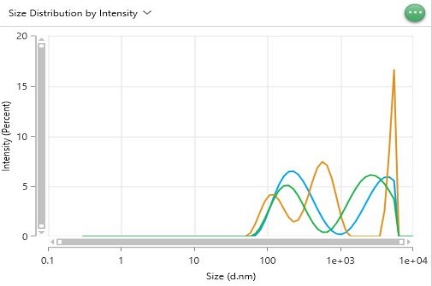

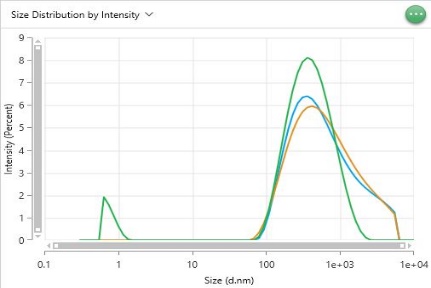

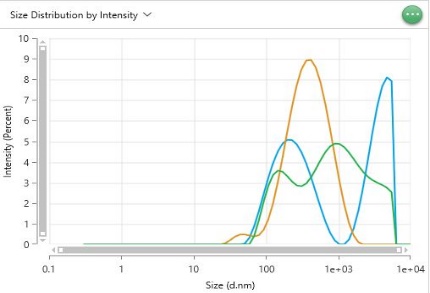

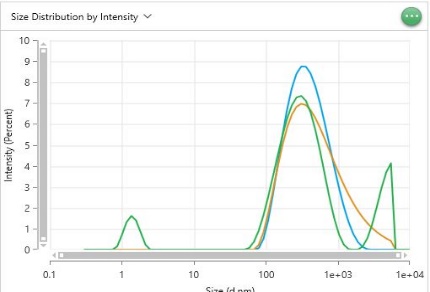

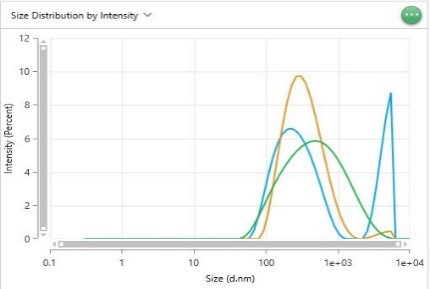


**B**

**10ms**

**20ms**

**30ms**

**500V**

**1 Pulse**

**2 Pulse**

**3 Pulse**

**Figure S2**. Particle size distribution plots of electroporated EVs at varying voltages, pulse numbers and pulse widths **(A)** before washing in DPBS and **(B)** after washing in DPBS.

**1000V**

**750V**

**Pulse Number**

**Pulse Width**

**500V**

**750V**

**1000V**

**Voltage**
